# Supplementary material for: Increased generalisation in trait anxiety is driven by aversive value transfer
Source: Commun Psychol. 2026 Feb 10;4:46. doi: 10.1038/s44271-026-00415-w (PMC13000330; doi:10.1038/s44271-026-00415-w)
Supplement: Supplementary file 2 — Supplementary Information [file 44271_2026_415_MOESM2_ESM.pdf]

Supplementary material

Title: Increased generalisation in trait anxiety is driven by aversive value transfer

Authors: Luianta Verra[1,2], Bernhard Spitzer[1,3], Nicolas W. Schuck[1,2,4*] and Ondrej Zika [1,4,5,6,7,8*]

Affiliations

1 Max Planck Institute for Human Development, Berlin, Germany
2 Institute of Psychology, Universität Hamburg, Hamburg, Germany
3 Faculty of Psychology, Technische Universität Dresden, Dresden, Germany
4 Max Planck UCL Centre for Computational Psychiatry and Aging Research, Berlin, Germany, and London, UK
5 Faculty of Psychology and Sports Science, Department of Psychology, Biological and Cognitive Neurosciences, Bielefeld University, Bielefeld, Germany
6 Centre for Psychiatry Research, Department of Clinical Neuroscience, Karolinska Institutet, & Stockholm Health Care Services, Region Stockholm, Stockholm, Sweden
7 Department of Clinical Psychology and Psychotherapy, Babeș-Bolyai University, Cluj-Napoca, Romania
8 School of Psychology, University College Dublin, Dublin, Ireland

These authors contributed equally: Nicolas W. Schuck, Ondrej Zika. Correspondence should be addressed to O.Z (email: ondrej.zika@pm.me) or to N.W.S (email: nicolas.schuck@uni-hamburg.de)

Supplementary Notes 1: Behavioural analyses

Perceptual titration

A titration procedure was used to create personalised stimulus spaces at target discriminability levels (60% and 80%). Final accuracy for high discriminability (mean = 79.88%, sd = 6.91%) and low discriminability (mean = 60.12%, sd = 7.53%) conditions closely matched the target accuracies and did not deviate from them (p=0.852 and p=0.839). Discriminability was manipulated by varying the perceptual distance of neighbouring stimuli κ. Accuracies correspond to average differences in κ of $\Delta_{\kappa,80\%}$=0.10 (sd = 0.024) for high and $\Delta_{\kappa,60\%}$=0.07 (sd = 0.023) for low discriminability conditions.

We next asked whether differences in TA were associated with final accuracy in the titration task by modelling final accuracy as a function of the fixed effects and interaction of TA and discriminability conditions. This model revealed no association of TA and accuracy (p = 0.165).

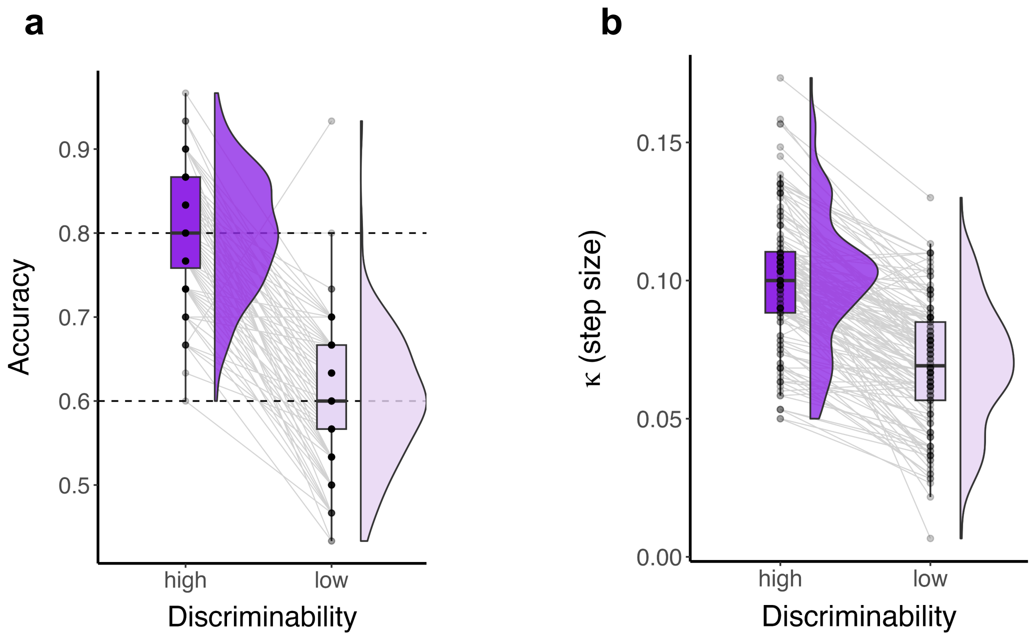

Supplementary Figure 1: Titration task results. a Accuracy for correctly recognizing two stimuli as different as a result of titration and b step size κ after titration grouped by discriminability level. The grey data points and lines represent participant means.

Similarity rating task

To assess changes in perception due to conditioning, similarity ratings (ranging from 0 = identical to 100 = completely different) between all pairs of neighbouring stimuli were collected before and after learning. Rated similarity reflected the two discriminability conditions introduced during titration, indicating that ratings significantly differed between the two perceptual discriminability conditions t(276)=5.43, p<0.001. In the low discriminability condition, participants perceived neighbouring stimuli as more similar compared to the high

discriminability condition, mean_low: 32.53, sd: 18.71; mean_high: 45.08, sd: 19.94. We fit a Beta regression with ratings as the dependent variable and block (before or after learning), distance of a stimulus pair from the CS+ and their interaction as fixed effects. We further included discriminability as a random intercept. The model revealed an effect of block, $\chi^2(1)=36.63$, $p<0.001$, indicating that participants perceived stimuli as more similar after (mean = 36.94, sd = 18.51) compared to before learning (mean= 40.67, sd = 19.08). The model further found a main effect of distance, indicating that participants' ratings increased with distance from the CS+, $\beta= 0.22$. This reveals that the stimulus space was not perceived linearly and that stimulus pairs were more discriminable at the edges of the space, $\chi^2(1)=228.32$, $p<0.001$, $\eta_p^2=0.09$ [0.08,0.10]. This distance based decrease in perceived similarity did not change over the course of conditioning, distance:pre-post: $\chi^2(1)=0.85$, $p=0.354$. Overall, similarity ratings provided additional evidence for successful perceptual manipulation during titration. We next asked whether differences in TA were associated with perceived similarity of stimuli by modelling similarity ratings as a function of the fixed effects and interaction of TA and discriminability conditions. This model revealed no association of TA and perceived similarity ($p = 0.056$).

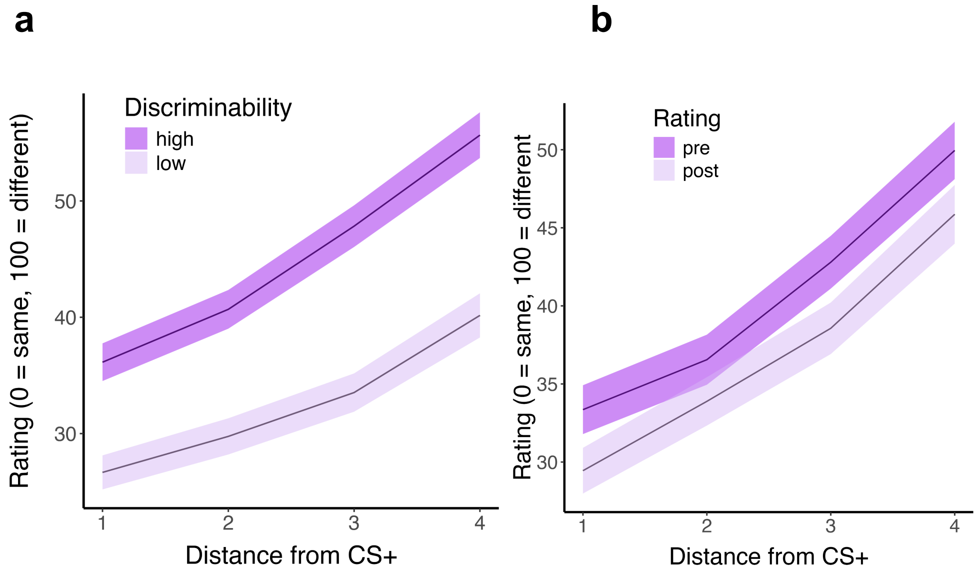

Supplementary Figure 2: **Similarity rating task.** a Similarity ratings as a function of distance of a stimulus pair from the CS+, where 0= identical and 100= completely different, split by perceptual discriminability condition. b Similarity ratings split by task block: pre (before conditioning) and post (after conditioning). Lines show mean ratings per group, shaded areas show the standard error of the mean.

Learning

Expectancy ratings reported after conditioning show that participants were able to learn the three reinforcement levels of 25%, 50% and 75% (mean_25 = 0.31, sd_25 = 0.18; mean_50=0.54, sd_50 = 0.14; mean_75 = 0.77, sd_75 = 0.11). One-sample t-tests against the target reinforcement level showed that participants overall overestimated outcome probabilities, but most strongly in the 25% condition, condition_25: $t(138)=4.1$, $p<0.001$,

condition_50: $t(139)=3.51$, $p<0.001$, condition_75: $t(137)=2.03$, $p = 0.04$. Ratings for the different reinforcement conditions differed significantly, $\chi^2(2)=813.81$, $p<0.001$, $\eta_p^2=0.9$ [0.87,0.92] between all levels, low-high: $z=-30.2$, $p<0.001$, d= -1.51[-1.61, -1.41]; low-mid: $z=-22.75$, $p<0.001$, d=-1.14[-1.24, -1.04]; high-mid: $z=24.19$, $p<0.001$, d=1.21[1.11,1.30]. To assess possible effects of TA we run a Beta regression model with the fixed effects and interactions of TA and reinforcement rate. The model found no significant modulation of learned contingency by anxiety (p =0.314).

Individual gradients by fitted rule Ω

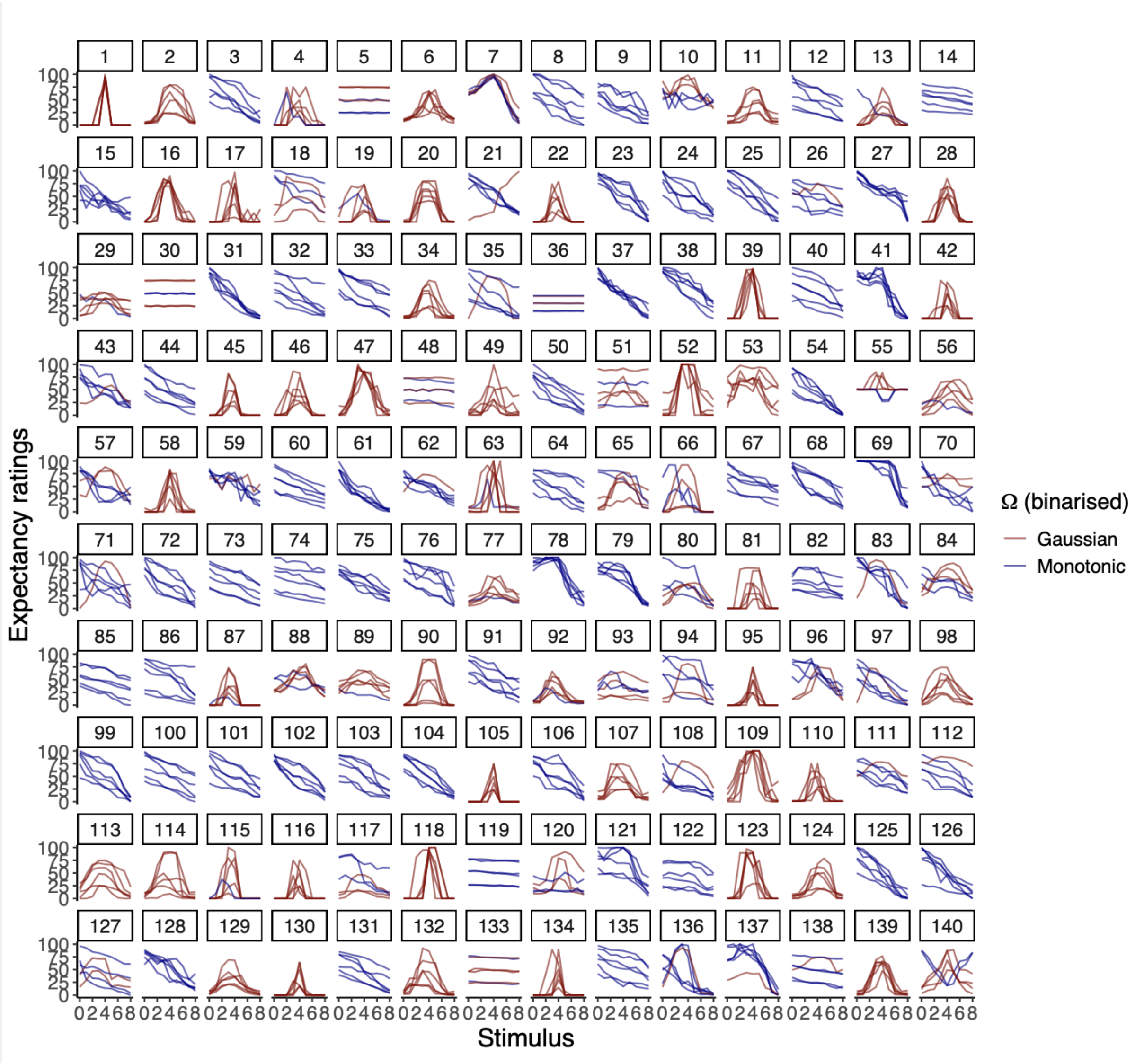

Supplementary Figure 3: **Individual gradient overview.** Overview of participant gradients for each of the 6 conditions varying in discriminability and reinforcement rate. Colour indicates the classification by the fitted parameter Ω, where Ω was binarised and indicates Monotonic (Ω <0.5, blue) or Gaussian-like (Ω >=0.5, red).

Supplementary Notes 2: Pattern/mechanism checks

Differences in learning

To assess whether the generalisation mechanisms and patterns related to differences in learning, we analysed CS+ ratings after learning (i.e., before generalisation) using a Beta GLM. We included reinforcement rate as a random intercept to account for differences between task conditions. The model found an effect of generalisation mechanism on ratings $\chi^2(1) = 8.06$, $p < 0.01$, $\eta_p^2 = 0.09$, CI = [0.0,0.25], with post hoc tests indicating that participants better fit by the value model had higher CS+ ratings after learning $mean_{value}$: 0.54, $mean_{perc}$: 0.50, value-perc: z=2.84, p = 0.004, d=0.08, CI=[0.01,0.15]. Using a similar approach on a subset of data fitted better by the value model we further found an effect of pattern, $\chi^2(1) = 5.47$, $p < 0.019$, $\eta_p^2 = 0.09$, CI = [0.0,0.24], with post-hoc tests indicating that the Gaussian pattern was associated with higher CS+ values after learning, $mean_{gauss}$:0.61, $mean_{monotonic}$:0.5, gaussian-monotonic: z=2.34, p = 0.019, d=0.09, CI=[0.01,0.17].

Differences in discriminability

Next, we tested whether the generalisation mechanism was associated with differences in stimulus discriminability by analysing accuracy in titration and perceived similarity ratings (i.e., similarity rating task). Similar to what we described for the learning ratings above, we first fit a LMM to accuracy data and a beta GLM to similarity ratings with generalisation mechanism as fixed effect while including the discriminability condition as a random intercept. The models found no effect of generalisation mechanism on either measure of perception (p=0.21and p=0.96). We proceeded with fitting models to the subset of the data best fit by the value generalisation model to assess the effect of the generalisation pattern on accuracy and similarity ratings. Neither model found an effect of pattern (p=0.96 and p=0.08), indicating that different generalisation mechanisms or the use of different generalisation patterns don't seem to be related to perceptual discriminability or subjective perceived similarity.

Supplementary Notes 3: Modelling

Parameter recovery

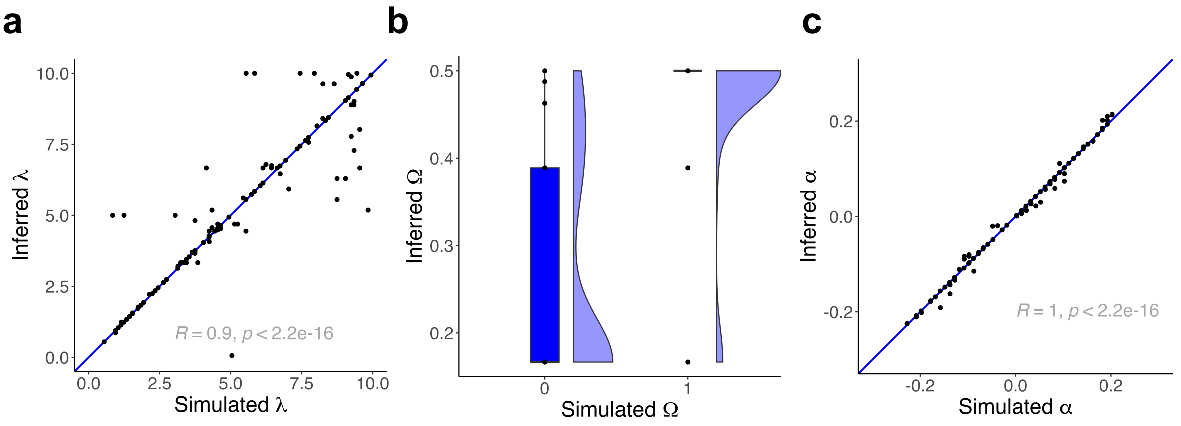

Supplementary Figure 4: **Parameter recovery.** Overview of parameter recovery results for parameters of the value model. a λ (generalisation width), b Ω (generalisation pattern),c α (offset).

Parameter correlations

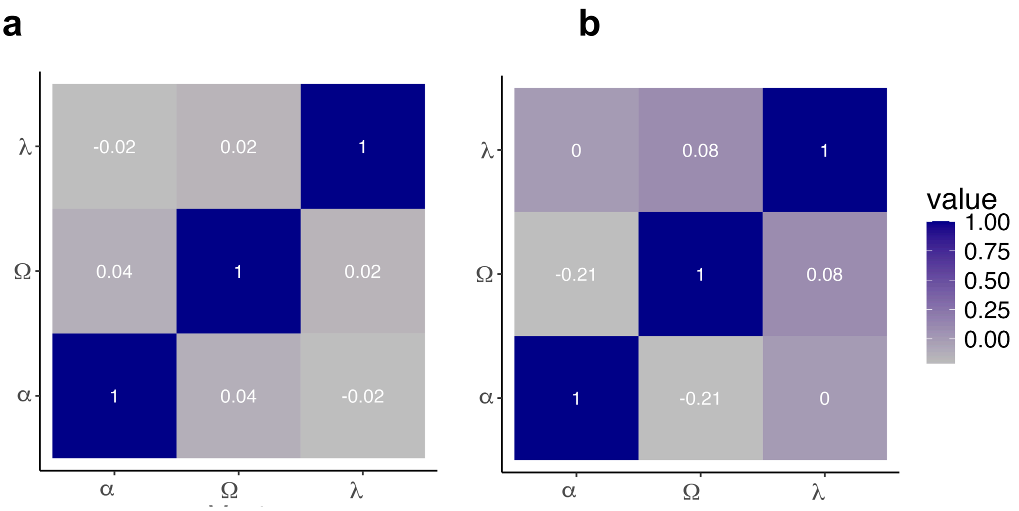

Supplementary Figure 5: **Parameter correlations.** Correlations of parameters for a simulated and b fitted data.

Parameter estimates

Model	Parameter	Ω<0.5 (LINEAR)			Ω >= 0.5 (GAUSSIAN)		
Perceptual	rho	0.59			0.21		
Value	RR	25%	50%	75%	25%	50%	75%
	lambda	2.82	3.42	3.98	2.28	2.24	2.47
	alpha	0.05	-0.02	-0.07	-0.02	-0.01	-0.07

Supplementary Table 1: **Parameter estimates for all models and experimental conditions**

Supplementary Notes 4: Demographics and Trait Anxiety scores

Sociodemographic Data	Age: **28 [18-40]**		
	Gender female/male: **47/93**		
Clinical and Cognitive Measures		mean	range

	**State-Trait Inventory for Cognitive and Somatic Anxiety**	**29.34**	**21-84**

Supplementary Table 2: **Questionnaire scores and demographics.**

We tested for gender differences in TA, finding no difference (p>0.346).

Supplementary Notes 5: Power Analysis

To determine our sample size and ensure sufficient power to detect an anxiety effect, we conducted a simulation-based power analysis using beta coefficients from a regression on a 40-participant exploratory pilot sample. The model included reinforcement rate, discriminability, generalisation pattern, and stimulus distance as fixed effects, their interactions with distance, and a random intercept for participants. Based on the anxiety*distance interaction ($\beta_{anxiety:distance}$ = 0.04), we simulated power for various sample sizes at α = 0.05, using the R simr package. Supplementary Figure 7 shows the resulting power estimates, indicating that a sample size of n = 134 achieves 85% power at alpha = 0.05.

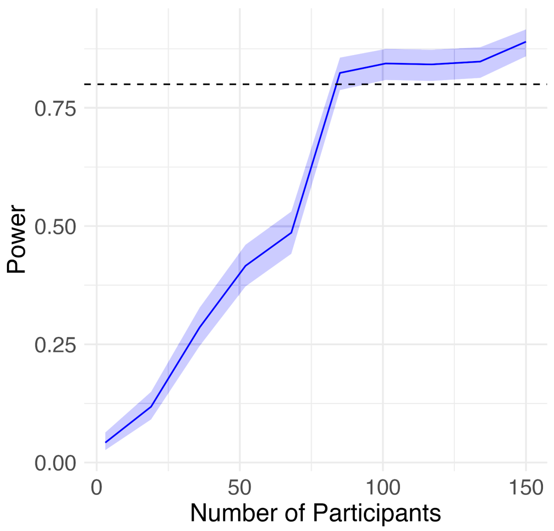

Supplementary Figure 7: **Power curve.** Power for different sample sizes based on simulations (n=500). Simulations use β- coefficients from a n=40 pilot sample and the dotted line represents power of 85%.

Supplementary Notes 6: Alternative models of generalisation

Hybrid model of generalisation

We fit a hybrid model that models both value generalisation and perceptual mistakes as an example of how perceptual mistakes and value generalisation interplay. Specifically, we add the probabilistic component of the perceptual model to the value generalisation model such that when participants misperceive a stimulus, the generalised value for the misperceived stimulus *q* is reported rather than the value for the presented stimulus *s*.

Similar to the perceptual model, the probability *P(q)* to perceive a stimulus *q* on a given trial depends on the perceptual discriminability of neighboring stimuli ρ and the distance of *q* from the presented stimulus *s*. *P(q)* decreases with increasing distance of a stimulus *q* from the presented stimulus *s*, following a multinomial distribution over stimuli given by

$$P(q|s) = \rho^{d_{|s-q|}} \quad \text{if } p \neq q$$
$$P(q|s) = 1 - \rho \quad \text{if } p = q$$

where $d_{|s-q|}$ corresponds to the distance between *q* and *s*.

Similar to the value model, the value for a specific stimulus V depends on a participants' generalisation function G and the generalisation tendency λ: $V = V_{CS+} * G_{\Omega}$. Analogous to the value model, the value function is specified as follows:

$$\text{If } \Omega = 1: \quad G_{\text{gaussian}} = \frac{2}{1 + e^{\frac{d_s^2}{\lambda}}}$$

$$\text{If } \Omega = 0: \quad \begin{cases} G_{s_{\{1:4\},\text{linear}}} = 1 + \left|1 - G_{s_{\{1:4\},\text{linear}}}\right| \\ G_{s_{\{5:9\},\text{linear}}} = G_{s_{\{5:9\},\text{gaussian}}} \end{cases}$$

d represents the distance of the perceived stimulus from the CS+. Analogous to the value model, the general tendency to over- or under-estimate value is specifying with a constant relative offset parameter $\alpha \in [-1, 1]$. Critically, the trial-wise predictions of the hybrid model correspond to the generalised values of the perceived stimulus *q*, not as in the value model the value of the shown stimulus *s*: $y = V_q + \alpha$

Model comparison

Model comparison of the perceptual and hybrid model shows similar results to when comparing the perceptual and value model (20% best fit by the perceptual model). Model comparison of the perceptual, value and hybrid model show an advantage of the value model over the full model, indicating that generalisation behaviour in the full model seems to be best captured by the value component. Here we note that the value and hybrid model don't fully recover and that this should be kept in mind when interpreting these results.

a **b**

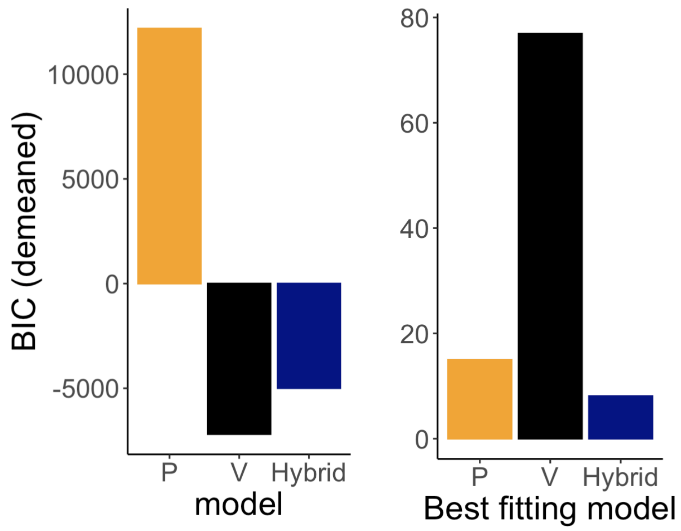

Supplementary Figure 8: **Model comparison and fits including Hybrid model.** a BIC scores of overall model fit for the perceptual, value and hybrid model, b percentage best fit by either model.

Binary choice model

We have tested the possibility of a binary choice mechanism i.e. participants generalise value using some function but their actions are binary. Specifically, we fit an alternative value model that additionally transforms beliefs stemming from value generalisation V_s before answering using a sigmoid transformation with fitted slope parameter *k.*

$$y_{transformed} = \frac{1}{1+e^{-k\,(V_s - 0.5)}}$$

This model fits better than the perceptual or value-based model in 11.67% of cases as can be seen in the figure below. When assessing if these gradients best fit by the alternative model were previously classified as value-based or perceptual we find that only 3.06% of these gradients were previously classified as perceptual. This shows that the sigmoid transformation improved the fit for those gradients that were already classified as value-based and that binary response distributions in our case seems to best be explained by a perceptual account.

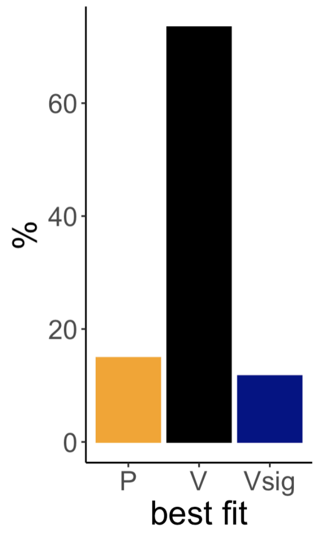

Supplementary Figure 9: % best fit by the perceptual model, value model and value model including a sigmoid transformation of values before response.

Learning model

We fit a simple Rescorla Wagner model to the learning data to assess whether participants differed in how fast they learned stimulus-outcome associations. We note that we only collected data halfway and at the end of learning and the estimated learning rates might be difficult to interpret. The model assumes that participants update their value estimates for the CS+ (V_{CS+}) on each trial *t+1* based on the prediction error $r_t - V_t$ between feedback *r* and prediction $V_{CS+,t}$ of the previous trial, weighted by a learning rate α. Initial $V_{CS+,t=0}$ is a free parameter and estimated.

$$V_{CS+,t+1} = V_t + \alpha * (r_t - V_{CS+,t})$$

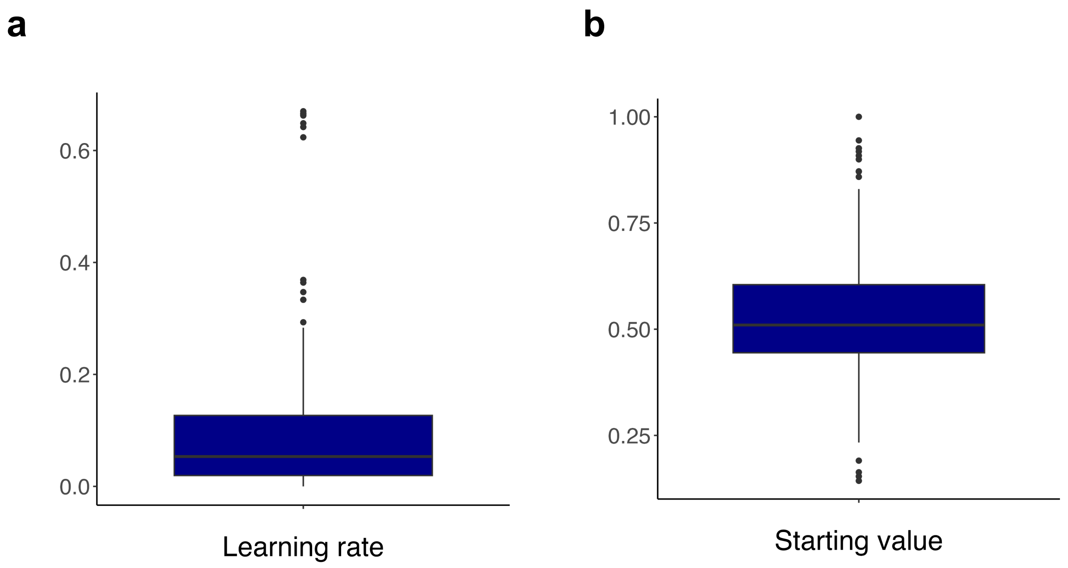

Supplementary Figure 10: **Parameters of Rescorla Wagner learning model.** a Estimated learning rates and b starting values for CS+ of the Rescorla Wagner learning model.

Association of learning rates with generalization and trait anxiety

We assessed whether learning rates are associated with the generalisation parameter λ, Pearson's r= -0.08, p=0.102, or anxiety scores, Pearson's r=0.13, p=0.112. This exploratory analysis revealed a negative association of lambda and learning rate, r =-0.24, CI = [-0.39,-0.08], p < 0.004, and no association of learning rates and anxiety scores.

Cross-validated model fits

We used k-fold cross-validation as an alternative way to evaluate model performance. In each fold, parameters were first estimated based on 80% of the data and tested on the remaining subset of 20%. This process was repeated five times, with each subset used once as the test set. To compare model fits, we report the root mean squared error (RMSE) averaged across the five folds and summed over conditions. Gradients best fit by the perceptual model increase by 9% compared to traditional fitting (from 13.1 to 22.5%). Overall, the results and conclusions remain unchanged.

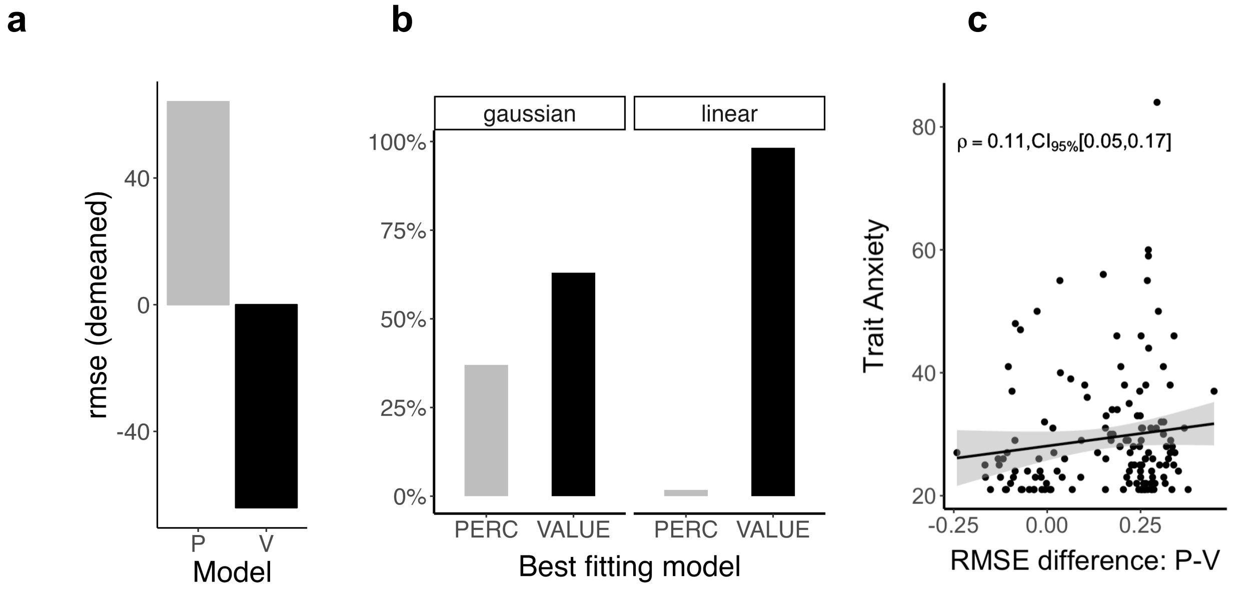

Supplementary Figure 11: **Model fit overview using cross-validation.** a Comparison of RMSE for the perceptual and value model. RMSE is averaged across folds and here represented as sum over all task conditions for each participant; b Percentage of gradients best fit by each model split by generalisation pattern; c Association of relative model fit and trait anxiety (right). Each dot represents one participant, the relative model fit was however based on fits per task condition. We show the correlation of TA and relative model fit with the 95% confidence intervals obtained through bootstrapping.

Relative model fit and anxiety

We repeated relative model fit analysis with cross-validated model fit results i.e. we related the difference in model fit to fixed effects and interactions of TA, reinforcement rate and discriminability. The model identified a positive relationship between TA and relative model fit β= 0.002, $\chi^2(1)$ = 6.24, p = 0.012, η_p^2= 0.02, CI = [0.00,0.08], indicating that TA was positively associated with a tendency towards better fit of the value model. This relationship was also evident in a simple correlation analysis (Pearson's r = 0.11; bootstrapped 95% CI = [0.05, 0.17]).
