# Supplementary material for: Increased generalisation in trait anxiety is driven by aversive value transfer
Source: Commun Psychol. 2026 Feb 10;4:46. doi: 10.1038/s44271-026-00415-w (PMC13000330; doi:10.1038/s44271-026-00415-w)
Supplement: Supplementary file 3 — Reporting Summary [file 44271_2026_415_MOESM3_ESM.pdf]

nature portfolio

Corresponding author(s): Nicolas Schuck, Ondrej Zika

Last updated by author(s): 25.06.2025

Reporting Summary

Nature Portfolio wishes to improve the reproducibility of the work that we publish. This form provides structure for consistency and transparency in reporting. For further information on Nature Portfolio policies, see our Editorial Policies and the Editorial Policy Checklist.

Statistics

For all statistical analyses, confirm that the following items are present in the figure legend, table legend, main text, or Methods section.

n/a	Confirmed	
☐	☒	The exact sample size (*n*) for each experimental group/condition, given as a discrete number and unit of measurement
☐	☒	A statement on whether measurements were taken from distinct samples or whether the same sample was measured repeatedly
☐	☒	The statistical test(s) used AND whether they are one- or two-sided<br>*Only common tests should be described solely by name; describe more complex techniques in the Methods section.*
☐	☒	A description of all covariates tested
☐	☒	A description of any assumptions or corrections, such as tests of normality and adjustment for multiple comparisons
☐	☒	A full description of the statistical parameters including central tendency (e.g. means) or other basic estimates (e.g. regression coefficient) AND variation (e.g. standard deviation) or associated estimates of uncertainty (e.g. confidence intervals)
☐	☒	For null hypothesis testing, the test statistic (e.g. *F*, *t*, *r*) with confidence intervals, effect sizes, degrees of freedom and *P* value noted<br>*Give P values as exact values whenever suitable.*
☒	☐	For Bayesian analysis, information on the choice of priors and Markov chain Monte Carlo settings
☐	☒	For hierarchical and complex designs, identification of the appropriate level for tests and full reporting of outcomes
☐	☒	Estimates of effect sizes (e.g. Cohen's *d*, Pearson's *r*), indicating how they were calculated

Our web collection on statistics for biologists contains articles on many of the points above.

Software and code

Policy information about availability of computer code

Data collection	JavaScript, JsPsych, LimeSurvey. Custom code is provided in associated repository.
Data analysis	R 4.2.1, Full list of packages and their version is available in associated repository in the renv.lock file.

For manuscripts utilizing custom algorithms or software that are central to the research but not yet described in published literature, software must be made available to editors and reviewers. We strongly encourage code deposition in a community repository (e.g. GitHub). See the Nature Portfolio guidelines for submitting code & software for further information.

Data

Policy information about availability of data

All manuscripts must include a data availability statement. This statement should provide the following information, where applicable:

- Accession codes, unique identifiers, or web links for publicly available datasets
- A description of any restrictions on data availability
- For clinical datasets or third party data, please ensure that the statement adheres to our policy

All data and code associated with the analyses are available on-line and full data and code availability statements are in the main manuscript.

Research involving human participants, their data, or biological material

Policy information about studies with human participants or human data. See also policy information about sex, gender (identity/presentation), and sexual orientation and race, ethnicity and racism.

Reporting on sex and gender	Participants were asked to self-report their sex. Information on gender was not collected. Sex or gender were not a variable of interest or covariate in any of the analyses. Participants' sex is included in the shared dataset which consists of 47 self-reported female and 93 self-reported male participants. Sex or gender was not considered in the study design as our hypotheses did not include any sex- or gender-based differences. We concluded no post-hoc sex- or gender-based analysis for lack of power.
Reporting on race, ethnicity, or other socially relevant groupings	NA
Population characteristics	Out of 140 participants, 47 were female, mean overall age was 28 years.
Recruitment	Participants were recruited on Prolific.
Ethics oversight	Ethics board of the Max Planck Institute for Human Development, Berlin

Note that full information on the approval of the study protocol must also be provided in the manuscript.

Field-specific reporting

Please select the one below that is the best fit for your research. If you are not sure, read the appropriate sections before making your selection.

☐ Life sciences ☒ Behavioural & social sciences ☐ Ecological, evolutionary & environmental sciences

For a reference copy of the document with all sections, see nature.com/documents/nr-reporting-summary-flat.pdf

Behavioural & social sciences study design

All studies must disclose on these points even when the disclosure is negative.

Study description	Quantitative experimental design. The data consisted of participants' scream expectancy ratings, same-different ratings and similarity ratings.
Research sample	Online sample recruited on Prolific. 140 participants completed all three sessions of the study, out of which 47 were female (overall mean age 28 years). Inclusion criteria on Prolific were: 95% Prolific approval rate, no current diagnosis of mental illness and no colour blindness. The sample was not assessed for representativeness. The goal was to recruit a random sample from the general public composed of individual without current psychiatric diagnosis or psychoactive medication.
Sampling strategy	Sample size was determined through a simulated power analysis using effect sizes of the main interactions of interest observed in an exploratory pilot sample of 40 participants, to reach power of 85% at alpha = 0.05.
Data collection	Data were collected online using Prolific for participant recruitment. The experiment ran on participants' web-browsers. Participants were required to wear headphones for the delivery of screams and sounds. Headphone use was assessed at the beginning of each session where the volume was further adjusted. Stimulus size was adjusted based on participants' viewing distance.
Timing	6.2023-01.2024
Data exclusions	Participants were required to pass a predefined accuracy threshold in session one to proceed with the remaining sessions. 33% did not continue. All participants that continued were included in the analyses (n=140).
Non-participation	No participants dropped out after having been selected to proceed with sessions 2 and 3.
Randomization	All participants completed all experimental conditions, in quasi-randomized order.

Reporting for specific materials, systems and methods

We require information from authors about some types of materials, experimental systems and methods used in many studies. Here, indicate whether each material, system or method listed is relevant to your study. If you are not sure if a list item applies to your research, read the appropriate section before selecting a response.

Materials & experimental systems

n/a	Involved in the study
☒	☐ Antibodies
☒	☐ Eukaryotic cell lines
☒	☐ Palaeontology and archaeology
☒	☐ Animals and other organisms
☒	☐ Clinical data
☒	☐ Dual use research of concern
☒	☐ Plants

Methods

n/a	Involved in the study
☒	☐ ChIP-seq
☒	☐ Flow cytometry
☒	☐ MRI-based neuroimaging

Plants

Seed stocks

Report on the source of all seed stocks or other plant material used. If applicable, state the seed stock centre and catalogue number. If plant specimens were collected from the field, describe the collection location, date and sampling procedures.

Novel plant genotypes

Describe the methods by which all novel plant genotypes were produced. This includes those generated by transgenic approaches, gene editing, chemical/radiation-based mutagenesis and hybridization. For transgenic lines, describe the transformation method, the number of independent lines analyzed and the generation upon which experiments were performed. For gene-edited lines, describe the editor used, the endogenous sequence targeted for editing, the targeting guide RNA sequence (if applicable) and how the editor was applied.

Authentication

Describe any authentication procedures for each seed stock used or novel genotype generated. Describe any experiments used to assess the effect of a mutation and, where applicable, how potential secondary effects (e.g. second site T-DNA insertions, mosiacism, off-target gene editing) were examined.
